# Supplementary material for: Genome-Wide Identification of Polyamine Oxidase (PAO) Family Genes: Roles of CaPAO2 and CaPAO4 in the Cold Tolerance of Pepper (Capsicum annuum L.)
Source: Int J Mol Sci. 2022 Sep 2;23(17):9999. doi: 10.3390/ijms23179999 (PMC9456136; doi:10.3390/ijms23179999)
Supplement: Supplementary file 1 [file ijms-23-09999-s001.zip › ijms-1853716-supplementary/Supplement figures.pdf]

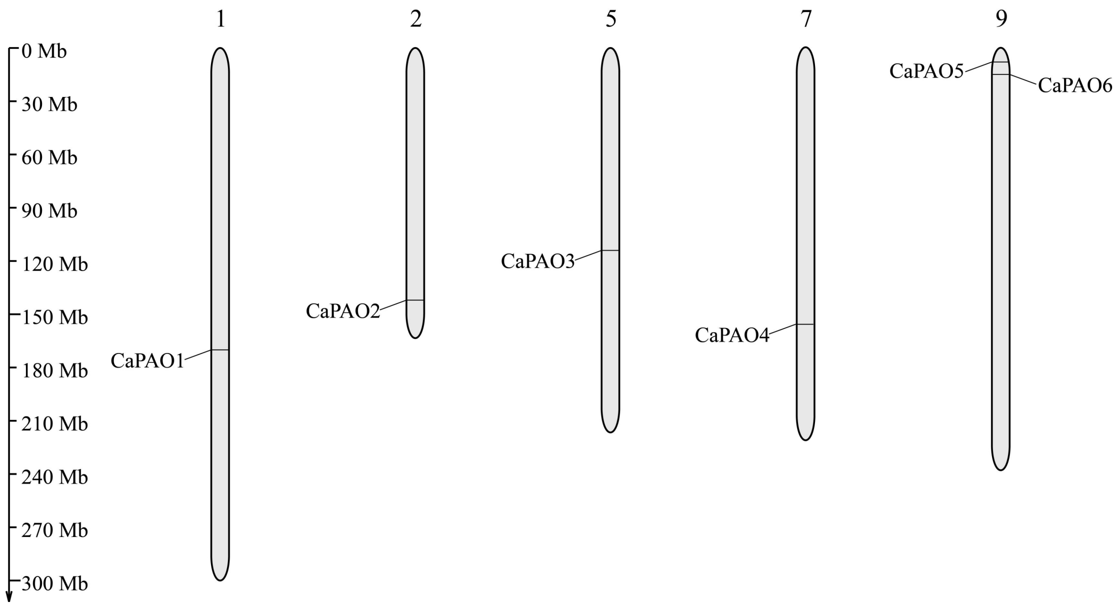

Figure S1. Chromosomal distribution of CaPAO genes in pepper.

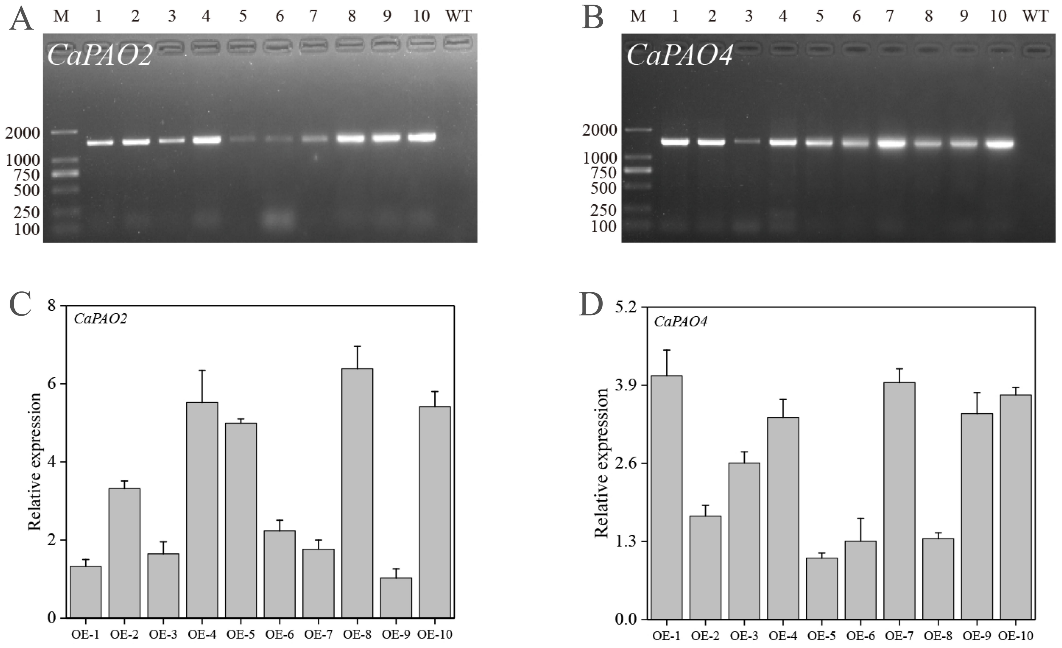

Figure S2. Identification and Expression Level analysis of *CaPAO2* and *CaPAO4* in transgenic *Arabidopsis*.

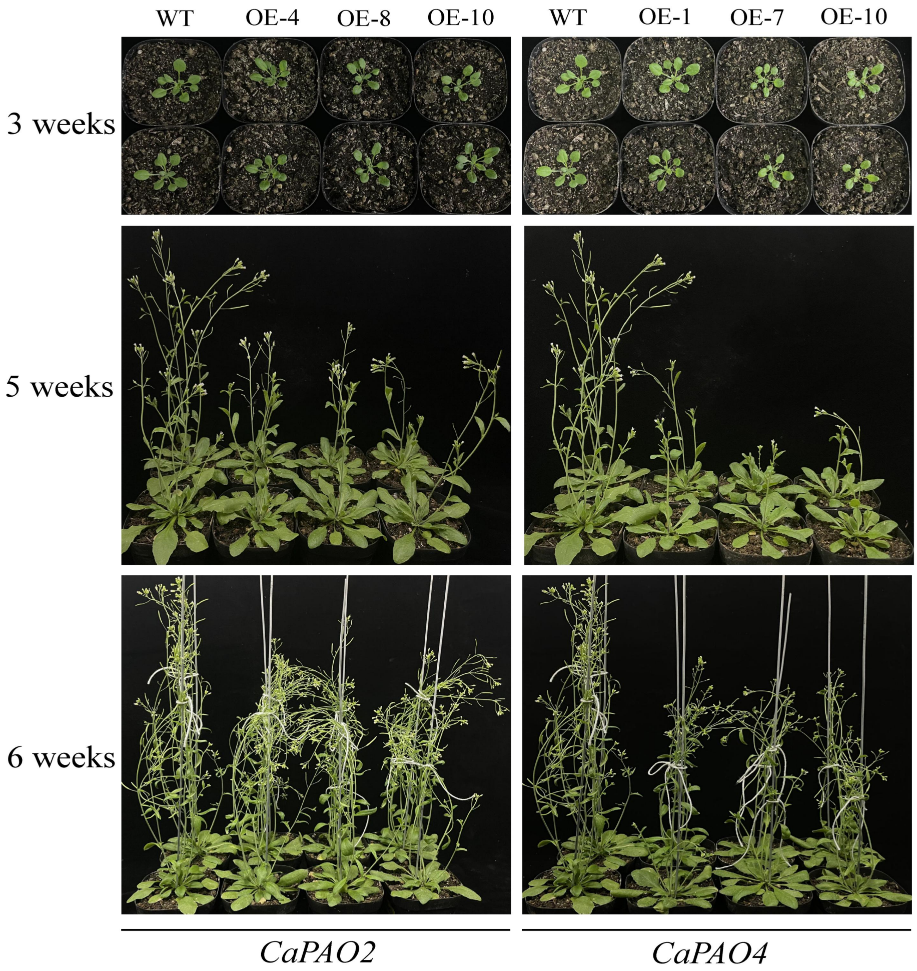

Figure S3. Phenotypic observation of WT and CaPAO2-OE and CaPAO4-OE lines under normal conditions.
