# Supplementary material for: Genome-Wide Identification of Polyamine Oxidase (PAO) Family Genes: Roles of CaPAO2 and CaPAO4 in the Cold Tolerance of Pepper (Capsicum annuum L.)
Source: Int J Mol Sci. 2022 Sep 2;23(17):9999. doi: 10.3390/ijms23179999 (PMC9456136; doi:10.3390/ijms23179999)
Supplement: Supplementary file 1 [file ijms-23-09999-s001.zip › ijms-1853716-supplementary/Supplement tables.pdf]

Table S1. Information of Cis-elements in promoters of pepper CaPAO genes.

Gene name	Site name	Sequence	Function
CaPAO5	TGACG-motif	TGACG	Methyl jasmonate responsive
CaPAO5	CGTCA-motif	CGTCA	Methyl jasmonate responsive
CaPAO1	CGTCA-motif	CGTCA	Methyl jasmonate responsive
CaPAO1	TGACG-motif	TGACG	Methyl jasmonate responsive
CaPAO2	TGACG-motif	TGACG	Methyl jasmonate responsive
CaPAO2	CGTCA-motif	CGTCA	Methyl jasmonate responsive
CaPAO4	CGTCA-motif	CGTCA	Methyl jasmonate responsive
CaPAO4	CGTCA-motif	CGTCA	Methyl jasmonate responsive
CaPAO4	TGACG-motif	TGACG	Methyl jasmonate responsive
CaPAO4	TGACG-motif	TGACG	Methyl jasmonate responsive
CaPAO5	TC-rich repeats	GTTTTCTTAC	Stress responsive
CaPAO5	TC-rich repeats	ATTCTCTAAC	Stress responsive
CaPAO2	TC-rich repeats	ATTCTCTAAC	Stress responsive
CaPAO1	TCA-element	TCAGAAGAGG	Salicylic acid responsive
CaPAO1	TCA-element	CCATCTTTTT	Salicylic acid responsive
CaPAO3	TCA-element	CCATCTTTTT	Salicylic acid responsive
CaPAO4	TCA-element	CCATCTTTTT	Salicylic acid responsive
CaPAO4	TCA-element	CCATCTTTTT	Salicylic acid responsive
CaPAO4	TCA-element	CCATCTTTTT	Salicylic acid responsive
CaPAO5	LTR	CCGAAA	Low-temperature responsive
CaPAO2	LTR	CCGAAA	Low-temperature responsive
CaPAO4	LTR	CCGAAA	Low-temperature responsive
CaPAO5	P-box	CCTTTTG	Gibberellin-responsive
CaPAO6	P-box	CCTTTTG	Gibberellin-responsive
CaPAO3	P-box	CCTTTTG	Gibberellin-responsive
CaPAO4	P-box	CCTTTTG	Gibberellin-responsive
CaPAO5	TATC-box	TATCCCA	Gibberellin-responsive
CaPAO2	MBS	CAACTG	Drought-inducibility
CaPAO3	MBS	CAACTG	Drought-inducibility
CaPAO5	TGA-element	AACGAC	Auxin-responsive
CaPAO6	TGA-element	AACGAC	Auxin-responsive
CaPAO1	TGA-element	AACGAC	Auxin-responsive
CaPAO1	TGA-element	AACGAC	Auxin-responsive
CaPAO3	TGA-element	AACGAC	Auxin-responsive
CaPAO3	TGA-element	AACGAC	Auxin-responsive
CaPAO3	AuxRR-core	GGTCCAT	Auxin-responsive
CaPAO5	ABRE	ACGTG	Abscisic acid responsive
CaPAO5	ABRE	ACGTG	Abscisic acid responsive
CaPAO5	ABRE	ACGTG	Abscisic acid responsive
CaPAO1	ABRE	ACGTG	Abscisic acid responsive
CaPAO1	ABRE	ACGTG	Abscisic acid responsive
CaPAO1	ABRE	ACGTG	Abscisic acid responsive

CaPAO2	ABRE	ACGTG	Abscisic acid responsive
CaPAO2	ABRE	ACGTG	Abscisic acid responsive
CaPAO2	ABRE	CACGTG	Abscisic acid responsive
CaPAO2	ABRE	ACGTG	Abscisic acid responsive
CaPAO6	GC-motif	CCCCCG	Anoxic inducibility
CaPAO6	GC-motif	CCCCCG	Anoxic inducibility

Table S2. Primers used for the qRT-PCR in pepper.

Primer name	Forward primer	Reverse primers
Q-CaPAO1	gtgttctccaaagcgacctc	atattggatcccgggtaagc
Q-CaPAO2	gctggaattatggctggtga	gagaggaaatgcagcttcga
Q-CaPAO3	cttcccttgttgagcccata	tttcatcgaaccccagaaag
Q-CaPAO4	tgtgattggtggtggaatgg	ctcggcctccaattctatcg
Q-CaPAO5	ccaattaggccgaaaagtca	aattgcgtcggtcttgaaac
Q-CaPAO6	tgttgacttgggtgcatcat	tctcgccaacctttgagact
Actin	tgtccatctgctctctgttg	caccccaagcacaataagac

Table S3. Primers used for the Validation of Transgenic Lines and qRT-PCR in *Arabidopsis*.

Primer name	Forward primer	Reverse primers
P-CaPAO2	atggaaatcaaggatgatgattctggc	tcacattcttgaaatctggagagga
P-CaPAO4	atggattctcaagtcaagagtaatcgc	ttacatacgggagatcaataaggggga
Q-CaPAO2	gctggaattatggctggtga	gagaggaaatgcagcttcga
Q-CaPAO4	tgtgattggtggtggaatgg	ctcggcctccaattctatcg
Q-*AtCOR15A*	gcagatggtgagaaagcgaa	ggcatccttagcctctcctg
Q-*AtRD29*	gccgagaaacttcagattgg	ccattcctcctcctcctttc
Q-*AtCOR47*	cagtgtcggagagtgtggtg	acagctggtgaatcctctgc
Q-*AtKIN1*	tggagctggagcacaaca	gacccgaatcgctacttgttc
AtActin2	taacagggagaagatgactcagatca	aagatcaagacgaaggatagcatgag

Table S4. Primers used for vector construction in pepper

Primer name	Primer sequences	Restriction endonuclease
GFP-CaPAO2-F	gcTCTAGAatggaaatcaaggatgatgattctgg	XbaI
GFP-CaPAO2-R	aaCTGCAGtcacattcttgaaatctggagaggaa	PstI
GFP-CaPAO4-F	gcTCTAGAatggattctcaagtcaagagtaatcgc	XbaI
GFP-CaPAO2-R	aaCTGCAGttacatacgggagatcaataaggggga	PstI
CaPAO2-OE-F	cagtCGTCTCacaacatggaaatcaaggatgatga	BsmBI
CaPAO2-OE-R	cagtCGTCTCatacatcacattcttgaaatctgga	Esp3I
CaPAO4-OE-F	cagtCGTCTCacaacatggattctcaagtcaagag	BsmBI
CaPAO4-OE-R	cagtCGTCTCatacattacatacgggagatcaata	Esp3I
CaPAO2-PE-F	cgGAATTCatggaaatcaaggatgatgattctgg	EcoRI
CaPAO2-PE-R	gacGTCTACtcacattcttgaaatctggagaggaa	SAI1
CaPAO4-PE-F	cgGAATTCatggattctcaagtcaagagtaatcg	EcoRI

CaPAO4-PE-R	gacGTCTACttacatacgggagatcaataagggga	SAI1

Note: F, forward primer; R, reverse primer; The upper case letters in the primer sequence are restriction endonuclease sites, and the underlined lower case letters are protective bases.
